# Perceptions of the Cardiologists and Oncologists: Initial Step for Establishing Cardio-Oncology Service

**DOI:** 10.3389/fcvm.2021.704029

**Published:** 2021-11-24

**Authors:** Hasan Ali Farhan, Israa Fadhil Yaseen

**Affiliations:** ^1^Scientific Council of Cardiology, Iraqi Board for Medical Specializations, Baghdad, Iraq; ^2^Baghdad Heart Center, Baghdad Teaching Hospital, Medical City, Baghdad, Iraq

**Keywords:** breast cancer, heart failure, hypertension, risk factor, hospitalization, mortality, online survey

## Abstract

**Background:** Over the last years, there was no established cardio-oncology service in Iraq and no firm data about the incidence of cardiovascular disease (CVD) among patients with cancer. As an initial step, we decided to conduct a national cardio-oncology online survey for cardiologists, oncologists, and their residents which would help us to understand the expected prevalence, problems, and readiness for collaboration between the two specialties.

**Objectives:** For evaluating the current national practice in the cardiology and oncology specialty fields and to identify the hidden gaps associated with the development or worsening of CVD among patients with cancer.

**Methods:** An online survey including 19-question for cardiologists/cardiology residents (CCRs) and 30-question for oncologists/oncology residents (OORs) about cardio-oncology service was sent to them including all Iraqi cities using Google document form during December 2020.

**Results:** The total number of responses was 164, mainly 62.2% from CCRs while 37.8% from OORs. Hypertension was the main baseline risk factor (71%). A 77.5% of CCRs prescribe cardiovascular drugs vs. 35.5% by OORs. About 76.5% of CCRs and 79% of OORs are facing difficulties in the management of patients with cancer with established CVD. CVD was the leading cause of both hospitalization (30.7%) and mortality (48.4%). About 62.8% of CCRs and 64.5% of OORs have an interest to work in cardio-oncology service.

**Conclusion:** Based on the perception of cardiologists and oncologists, CVD is the main cause of hospitalization and mortality among patients with cancer. High interest among CCRs and OORs to work in cardio-oncology service. Positive initiatives are available to take the action plan in this emerging field.

## Introduction

Cardio-oncology is an emerging specialty and service with a team-based approach that includes cardiologists, oncologists, and hematologists working collaboratively for optimizing cardiovascular risk stratification, prevention, and treatment for all patients with cancer and survivors to guide best practice by bridging the gaps in knowledge and needs including supporting patients with cancer to improve continuing of their cancer therapies without interruption by the cardiovascular disease (CVD) (1–4). First cardio-oncology service emerged in 2010 (1, 4), therefore, such essential service was not available worldwide including Iraq. A descriptive study in the United Kingdom including data over 5-year activity from cardio-oncology service reported that baseline CVD and myocardial toxicity are higher than that was documented in previous studies (5). This is why continuing national and international research in the cardio-oncology field is of high importance for a better understanding of the current practice and optimization of cardio-oncology services. In 2019, the Iraqi Cardio-Oncology Program was founded by a senior consultant cardiologist and his mentee the cardiology clinical pharmacist to support the initiation of cardio-oncology services and as an initial step for evaluating the current national practice and uncovering the hidden gaps associated with the development or worsening of CVD especially during a very critical time of COVID-19 era, therefore, it was decided to conduct an online survey. An online survey has several advantages, including low costs, real-time access, not time-consuming, and respondents may be more willing to participate and had documented the need to improve the care of patients (2, 6).

## Methods

### Ethical Approval

The study was approved by the Department of Continuing Medical Education at the Iraqi Board for Medical Specializations.

### Study Design

An online survey link including 19 questions for cardiologists/cardiology residents (CCRs) and 30 questions for oncologists/oncology residents (OORs) about cardio-oncology service was sent to participants who are working in cardiology and oncology sites in all 18 Iraqi cities using Google document form in December 2020. The link was shared with the participants by sending a message on WhatsApp or Viber applications for responding to the survey voluntarily. The message was sent either individually or by sharing it on WhatsApp or Viber groups for cardiologists, oncologists, and their residents.

### Survey Questionnaires

Questions were divided into three categories: (1) about demographics of participants, (2) questions about current practice, and (3) questions about their opinions about cardio-oncology service. Some of the questions in categories two and three were directed only to oncologists [for OORs] related to baseline patients' characteristics and referral. One question was directed only to cardiologists (for CCRs). Other questions were directed to both cardiologists and oncologists. The single choice answer was used, including the “other” option to add unavailable suitable answers of the participants. Multiple choice answers were used for the cardio-oncology team question. Age was typed by the participants. Collected data were filled in excel for double check and analyzed in excel using numbers, percentages, average, and SD for the continuous variables.

### Statistical Analysis

The statistical analysis was performed using Excel for Mac, Version 15.13.3.

## Results

### Demographics of Participants

Most of the responders were male and specialists; cardiologists and oncologists, with age younger than 66 years old. Results are available in Table 1.

**Table 1:** Demographics of participants.

		**Cardiology** **Total (*N* = 102)**	**Oncology** **Total (*N* = 62)**
Age (years)		43 ± 6	38 ± 6
		33–65	27–52
Gender	Male	98.0 (100)	59.7 (37)
	Female	2.0 (2)	40.3 (25)
Specialist		63.7 (65)	64.5 (40)
Residents		36.3 (37)	35.5 (22)

*Values are mean ± SD, % (n), or range*.

### Questions About Current Practice Regarding Cardio-Oncology Patients

**Figure 1:**
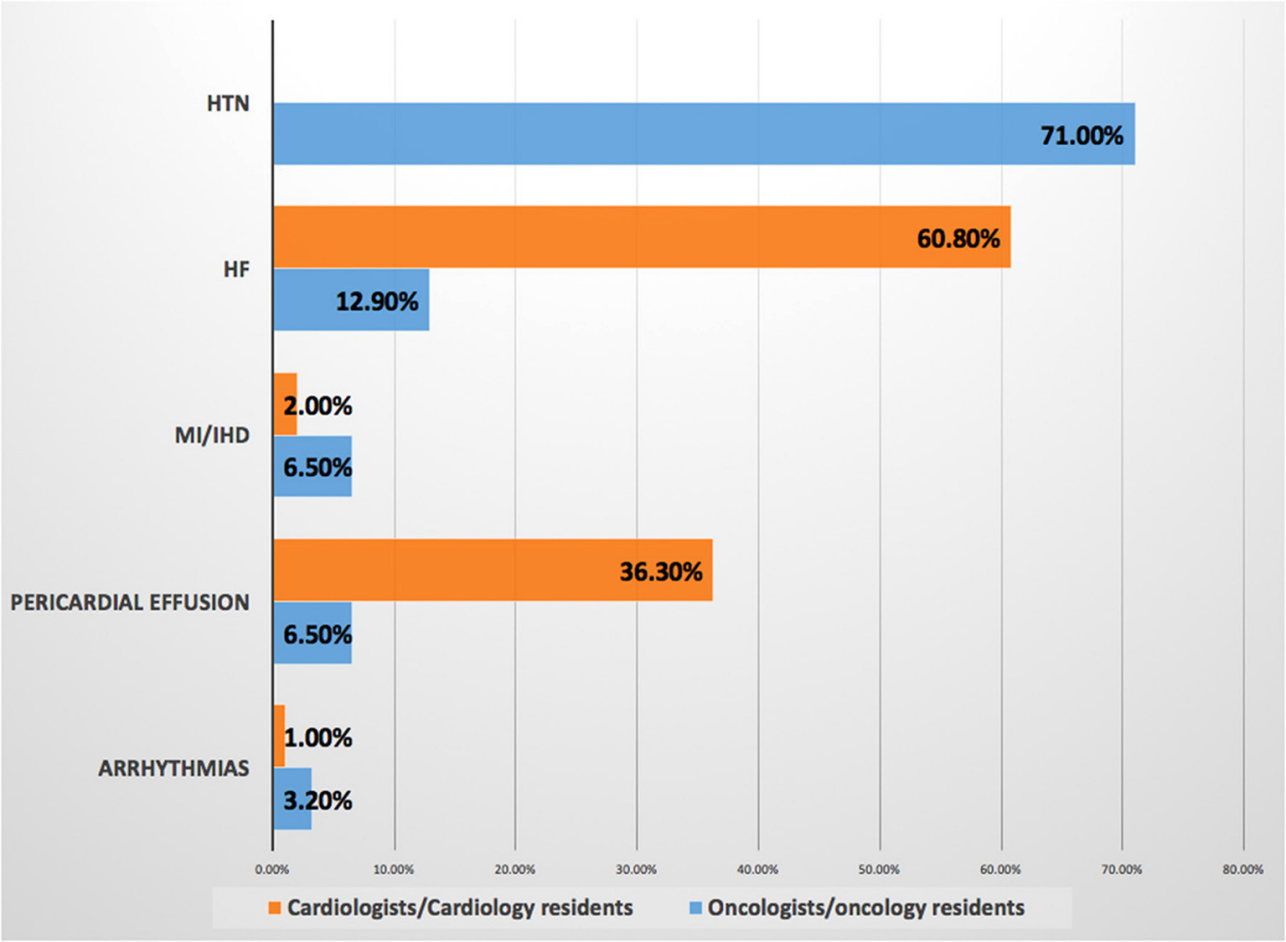
Most common baseline cardiovascular disease among patients with cancer. Prevalence of baseline cardiovascular disease, hypertension is the most common risk factor according to responses of the oncologists/oncology residents. HF, heart failure; HTN, hypertension; IHD, ischemic heart disease; MI, myocardial infarction.

**Figure 2:**
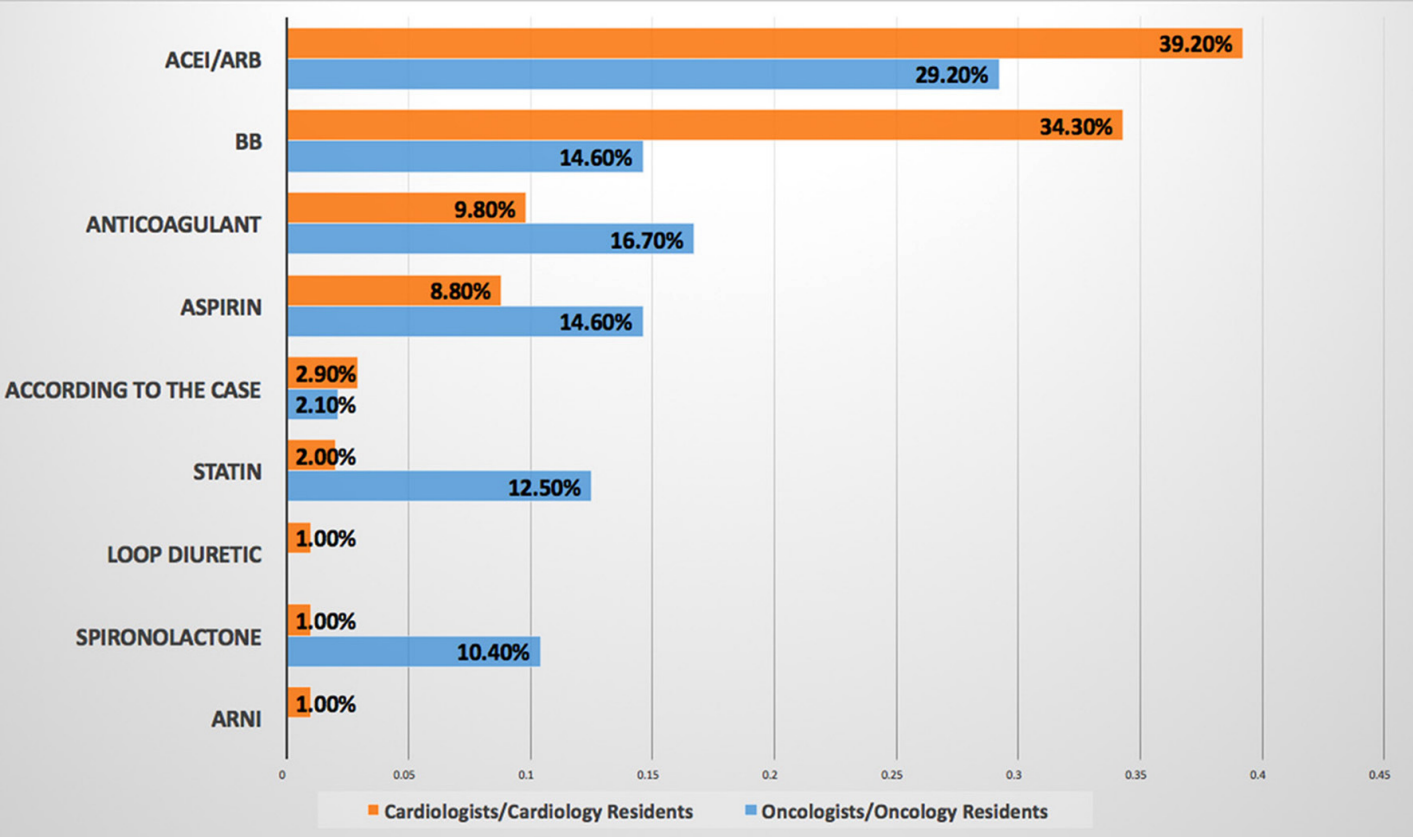
Most commonly prescribed cardiovascular drug for patients with cancer. ACEI/ARB are the most commonly prescribed cardiovascular drug for patients with cancer by both cardiologists/cardiology residents and oncologists/oncology residents. ACEI, angiotensin-converting enzyme inhibitor; ARB, angiotensin receptor blocker; BB, beta-blocker; ARNI, angiotensin receptor-neprilysin inhibitor.

**Figure 3:**
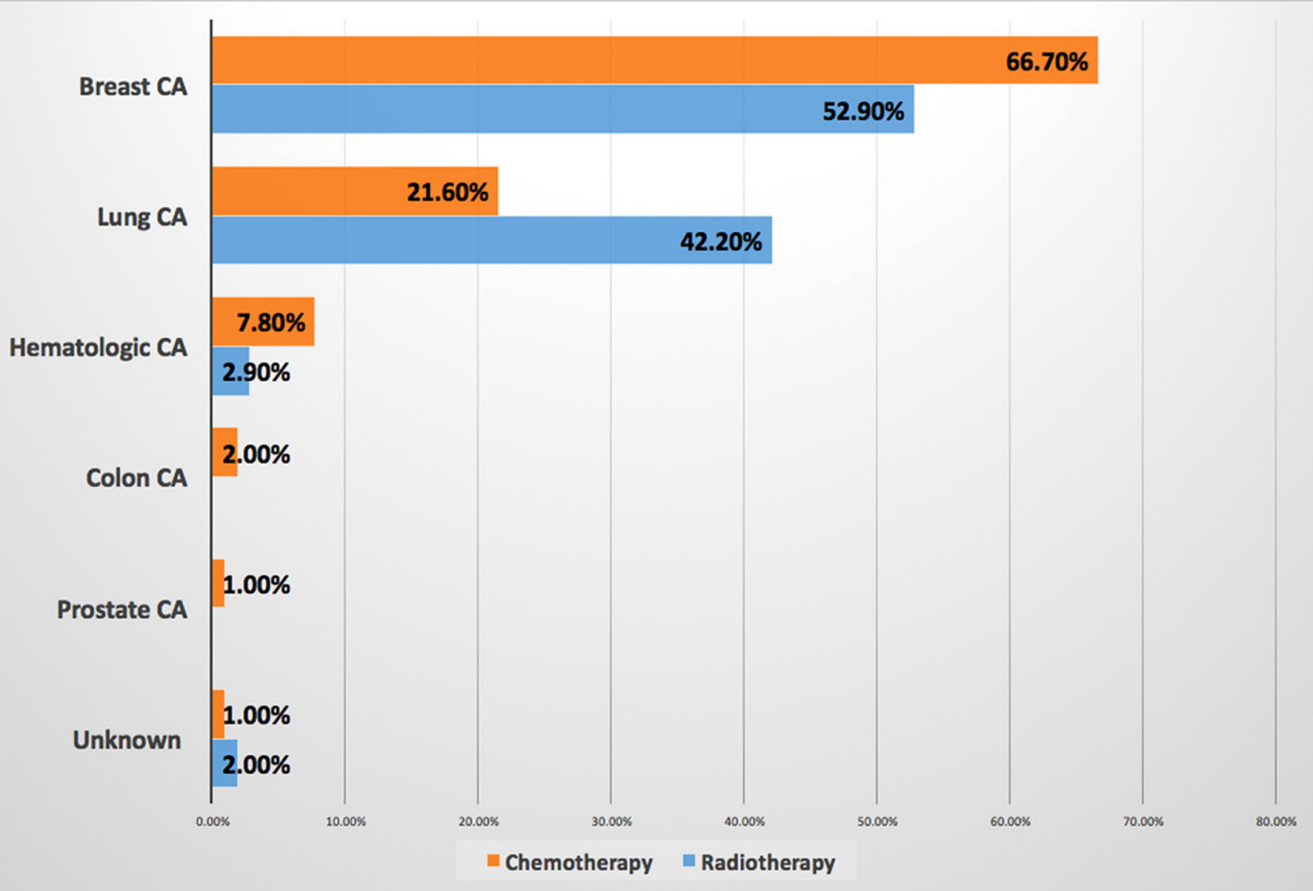
Most cancer types associated with cancer therapy-induced cardiovascular disease. Breast cancer is the most common malignancy associated with CVD induced by both chemotherapy and radiation therapy. CA, cancer; CVD, cardiovascular disease.

**Figure 4:**
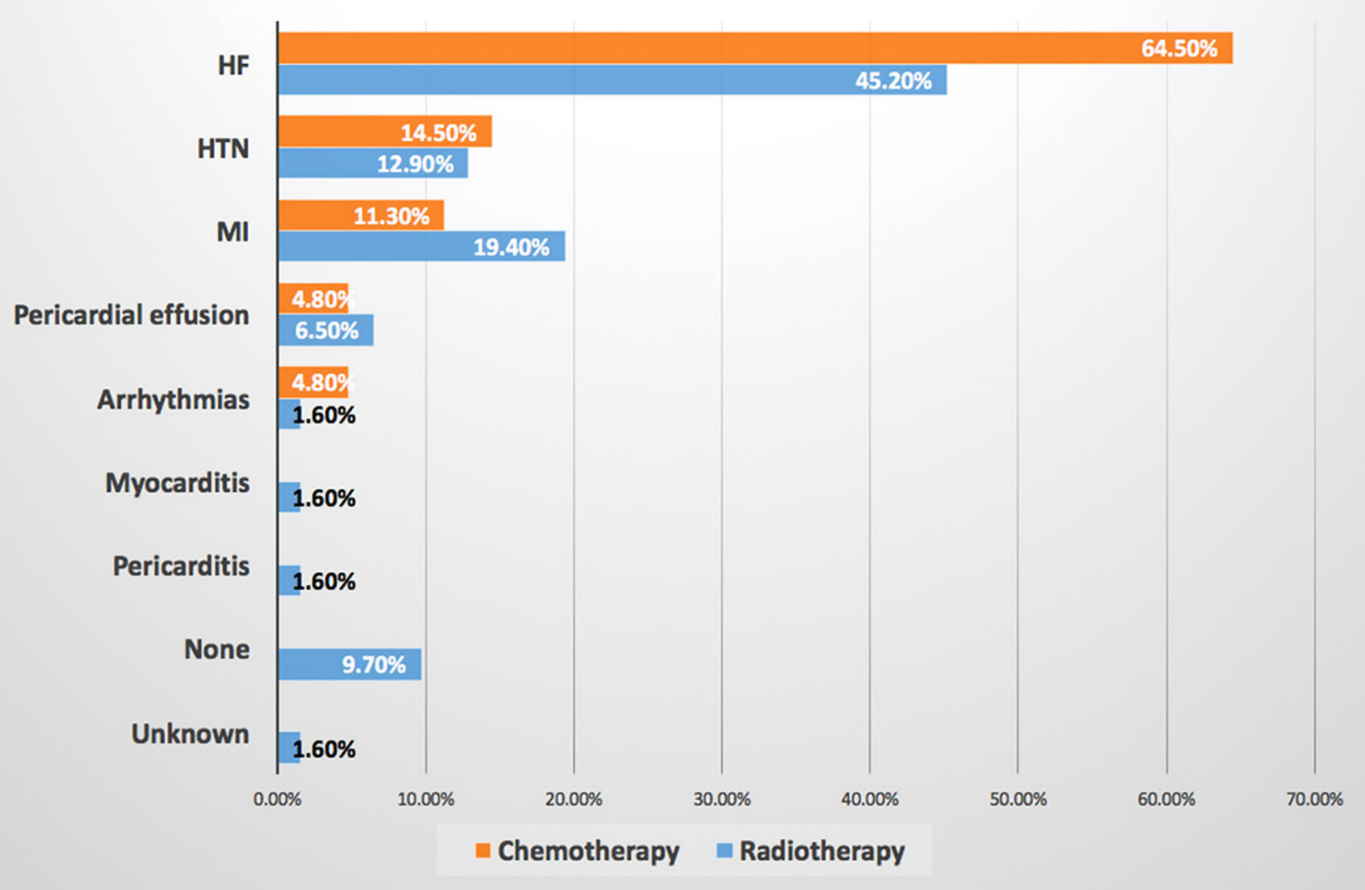
Most common cardiovascular disease induced by chemotherapy and radiotherapy. HF is the most common CVD induced by both chemotherapy and radiation therapy. HF, heart failure; HTN, hypertension; MI, myocardial infarction.

**Figure 5:**
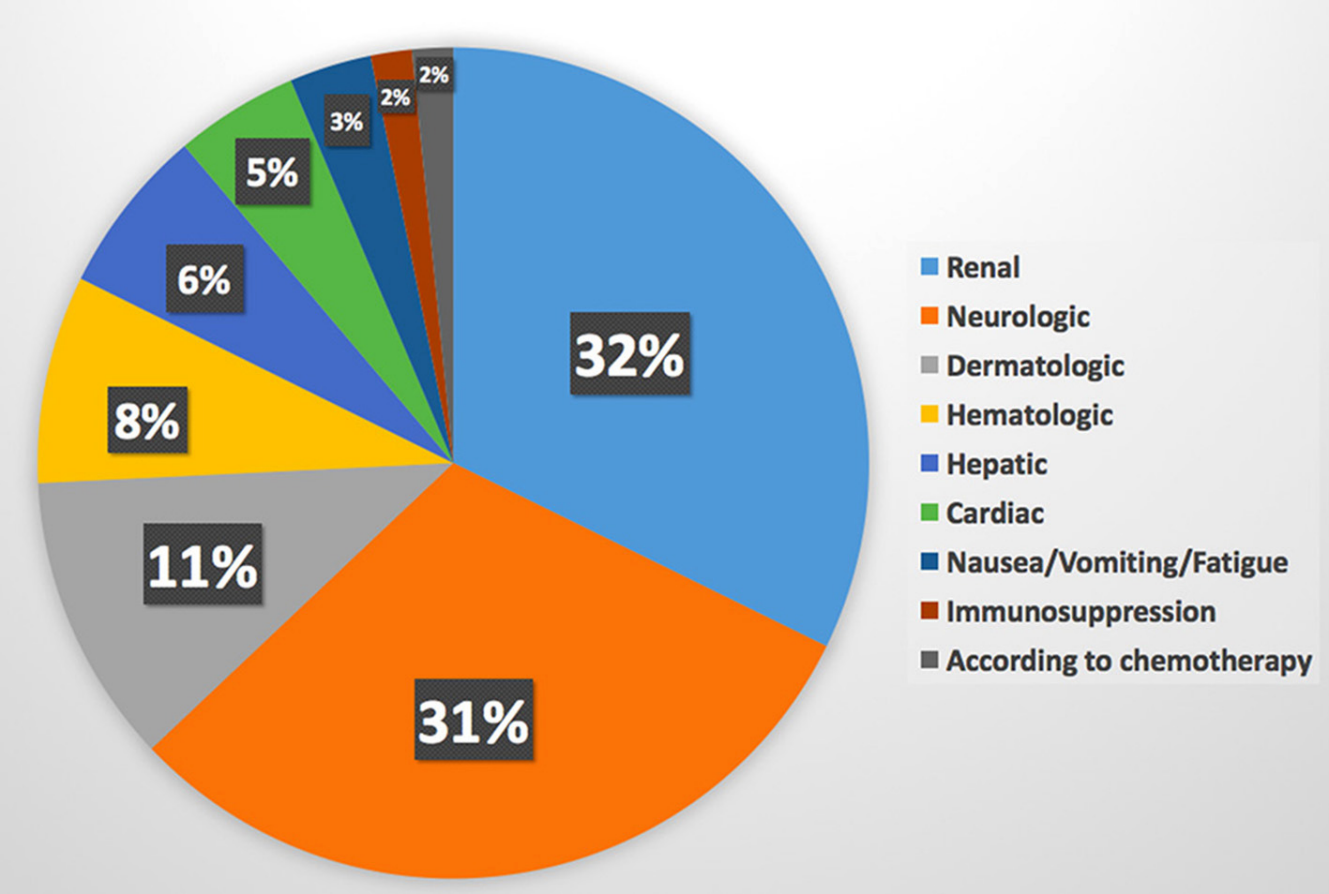
Most common complications of chemotherapy. Cardiovascular disease is the sixth complication of chemotherapy.

**Figure 6:**
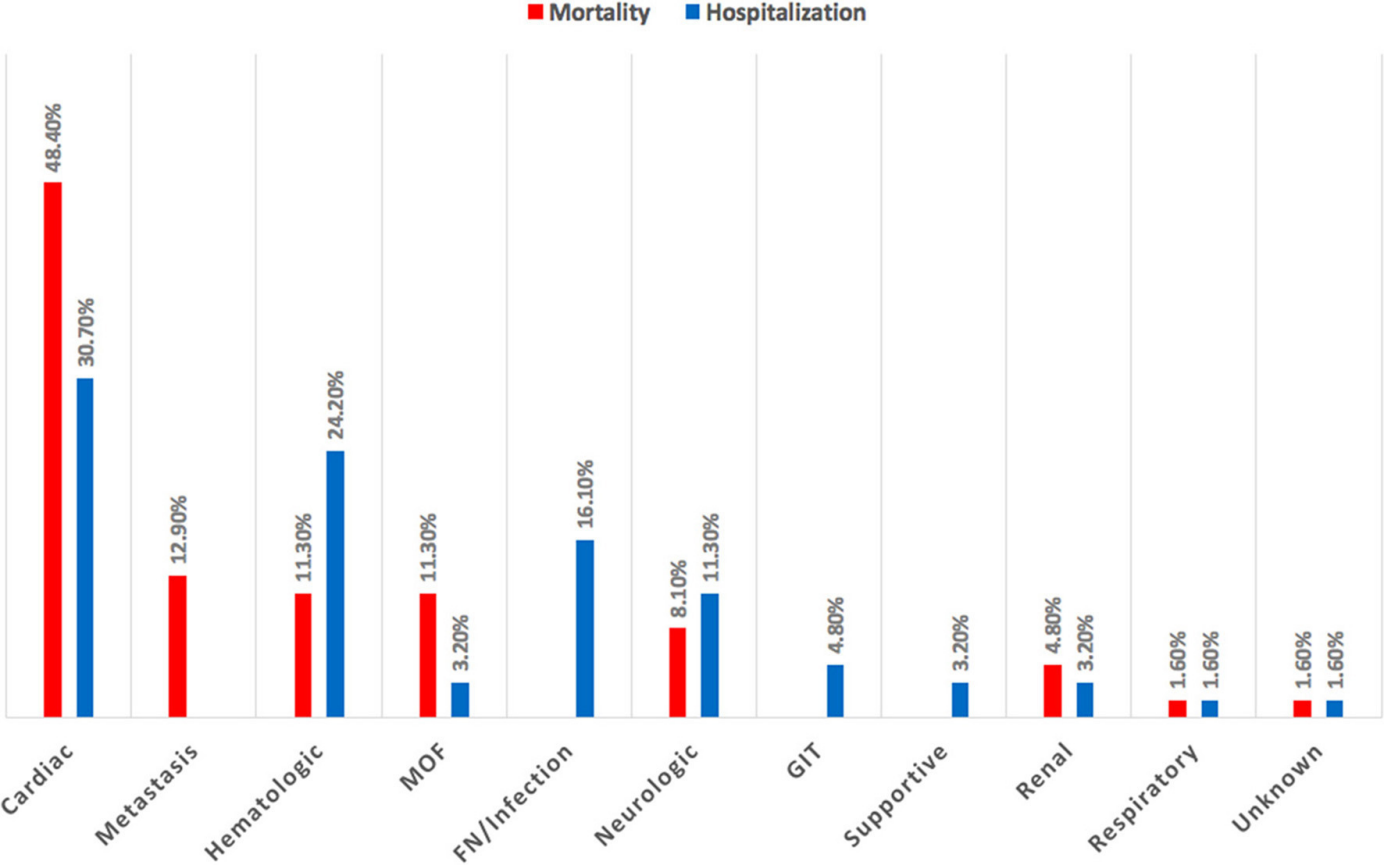
Main medical cause for hospitalization and mortality among patients with cancer. Cardiovascular disease is the leading cause of hospitalization and mortality among patients with cancer. GIT, gastrointestinal tract; MOF, multi-organ failure.

**Figure 7:**
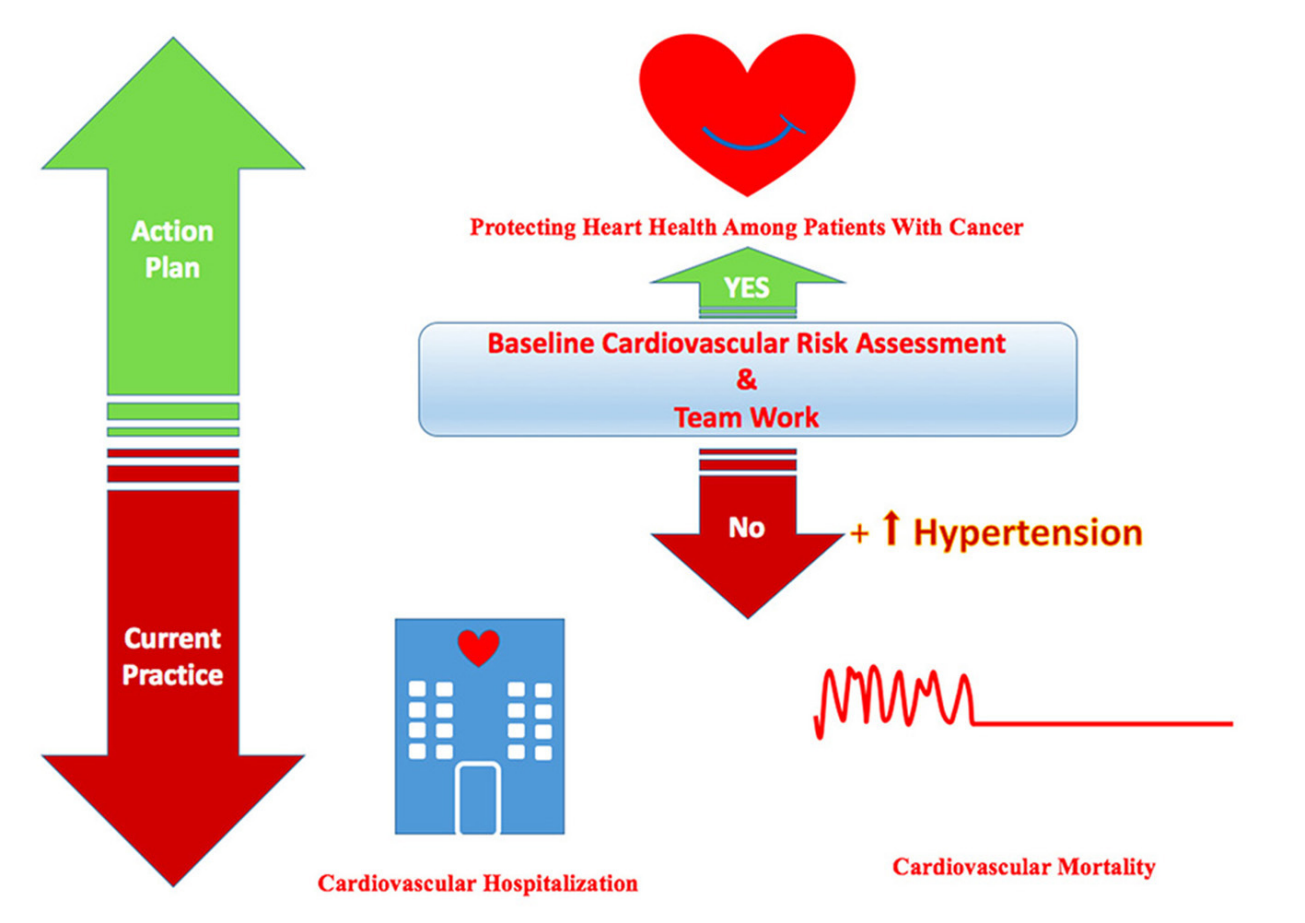
Central illustration showing the current practice and the required action plan to protect heart health among patients with cancer. Hypertension is the most common baseline cardiovascular risk factor among cancer patients according to the current practice which can be the leading cause of cardiovascular hospitalization and mortality. The action plan is required to protect the heart health of cardio-oncology patients by baseline cardiovascular risk assessment and teamwork.

Hypertension (HTN) is the most common baseline CVD among patients with cancer according to the OOR experiences (71%), while heart failure (HF) was the most common one as a result of the CCR responses (60.3%). The results of these questions are available in (Table 2 and Figures 1–7).

**Table 2:** Questions about current practice regarding patients with cardio-oncology.

1. What is the most common cardiac disease in patients with cancer at presentation?	responses of OORs showed that hypertension is the most common cardiac disease accounting for 71%, followed by HF 12.9%, pericardial effusion 6.5%, ischemic heart disease 6.5%, and arrhythmia 3.2%. While CCRs' responses were HF 60.8%, pericardial effusion 36.3%, arrhythmias 2%, and ischemic heart disease 1% (Figure 1).
2. What is the most common type of cancer associated with CVD?	Breast (71.6%), lung (17.7%), and hematologic (5.9%), and colon cancers were the most common types according to the response of CCRs. OORs chose breast (72.6%), lung (21%), prostate (3.2%), and colon cancer (1.6%) as the most common type while (1.6%) had no idea.
3. How often do you check the anticancer agents (question for CCRs)/cardiovascular drug (question for OORs) of patients with cancer with cardiac disease when presenting to you whether at private clinic or hospital?	The response to the option [Drugs were checking as required depending on the symptoms of the patients] was chosen by 48.1% of CCRs and by 82.3% of OORs. Checking drugs each visit was recorded by 46.1 and 9.7% of CCRs and OORs, respectively. While checking at first visit only was the response of (2.9%) of CCRs and (8.1%) by OORs. Among CCRs (2.9%) mentioned that they never check the cancer therapy of the patients.
4. Do you prescribe cardiovascular drugs for patients with cancer?	Only (77.5%) of CCRs mentioned that they are prescribing cardiovascular drugs for cancer patient who are (at risk) of developing the cardiac disease, while (35.5%) of OORs mentioned they are prescribing cardiovascular drugs for patients with cancer.
5. Which is the most cardiovascular drug do you prescribe for patients with cancer?	Responses from all cardiology participants and 48 (77.4%) oncology participants showed that ACEI/ARB are the most frequent cardiovascular drugs to be prescribed, followed by beta-blocker by CCRs and anticoagulant by OORs (Figure 2).
6. Do you face any difficulty in taking management action plan among patients with cancer with CVD?	The difficulty was faced among (76.5%) of CCRs when a patient with cancer refers to them to decide to continue or withhold chemotherapy/radiotherapy due to cardiac disease, and (79%) of OORs are facing difficulty in planning management strategy of cancer in a patient with cardiac disease.
7. What's the most common cancer type presents to you suffering from cardiac disease due to chemotherapy and radiotherapy?^*^	Breast cancer is the most common cancer type associated with CVD induced by both chemotherapy (66.7%) and radiation therapy (52.9%). Results are available in Figure 3.
8. What's the most common cardiac disease due to chemotherapy and radiotherapy is developed among your patients?^**^	HF is the most common CVD induced by chemotherapy (64.5%) and radiation therapy (45.2%).Results are available in Figure 4.
9. Do you face any interaction between cardiovascular drugs and anticancer?^**^	About 40.3% of OORs' responses reveal that they are facing interaction between cardiovascular drugs and anticancer therapy.
10. What's the most complications of chemotherapy do you face in patients with cancer?^**^	Renal impairment is the most common complication of chemotherapy (32%) while CVD is the sixth one (5%). Results are available in Figure 5.
11. Most of patients with cancer presented initially before starting anticancer or radiotherapy categorized under which group? ^**^	Most of the responses (61.3%) categorized patients to have baseline cardiovascular risk factors, while (21%) under the category of established cardiac disease (i.e., history of CVD). Finally, (17.7%) of responses showed that patients with cancer are presented most commonly with no history of CVD.
12. What's the main medical cause for hospitalization and mortality among patients with cancer?^**^	CVD is the leading cause of hospitalization (30.7%) and mortality (48.4%) according to the response of OORs as shown in Figures 6, 7.
13. Monthly, how many patients with cancer with cardiac disease do deal with approximately?^**^	An average of 10 patients monthly.
14. Do you refer all newly diagnosed patients with cancer to cardiologist for baseline cardiac evaluation before initiating anticancer or radiotherapy?^**^	A 62.9% of OORs refer newly diagnosed patients for cardiac evaluation, 37.1% of them do not send their patients.
15. For patients with cancer with cardiac symptoms (dyspnea, palpitation, etc.) do you send the patient for ECG/echocardiography only or for cardiac referral too?^**^	The majority of responses (79%) indicated that OORs sends such patients for ECG/echocardiography together with cardiac referral, (11.3%) only send for ECG/echocardiography, while (9.7%) send patients directly for a cardiac referral without checking ECG/echocardiography.
16. Do you refer only newly diagnosed patients with cancer with known cardiac disease to cardiologist for baseline cardiac evaluation before initiating anti-cancer or radiotherapy? Or do you refer all newly diagnosed patients with cancer?^**^	Most of the responders (54.8%) send all patients for baseline cardiac evaluation, however, (37.1%) send only patients with a known history of cardiac disease. The rest of the responders (8.1%) mentioned that they send patients as required, all patients with breast cancer, patients with CVD or risk factors if they will use cardiotoxic chemotherapy, or when using known cardiotoxic agents in all patients with cancer.

* *Indicating this question was directed only for CCRs*.

** *Indicating this question was directed only for OORs*.

*ACEI/ARB, angiotensin-converting enzyme inhibitor/angiotensin receptor blocker; CCRs, cardiologists/cardiology residents; OORs, oncologists/oncology residents; CVD, cardiovascular disease; ECG, electrocardiogram; HF, heart failure*.

### Questions About Their Opinions About Cardio-Oncology Service

**Figure 8:**
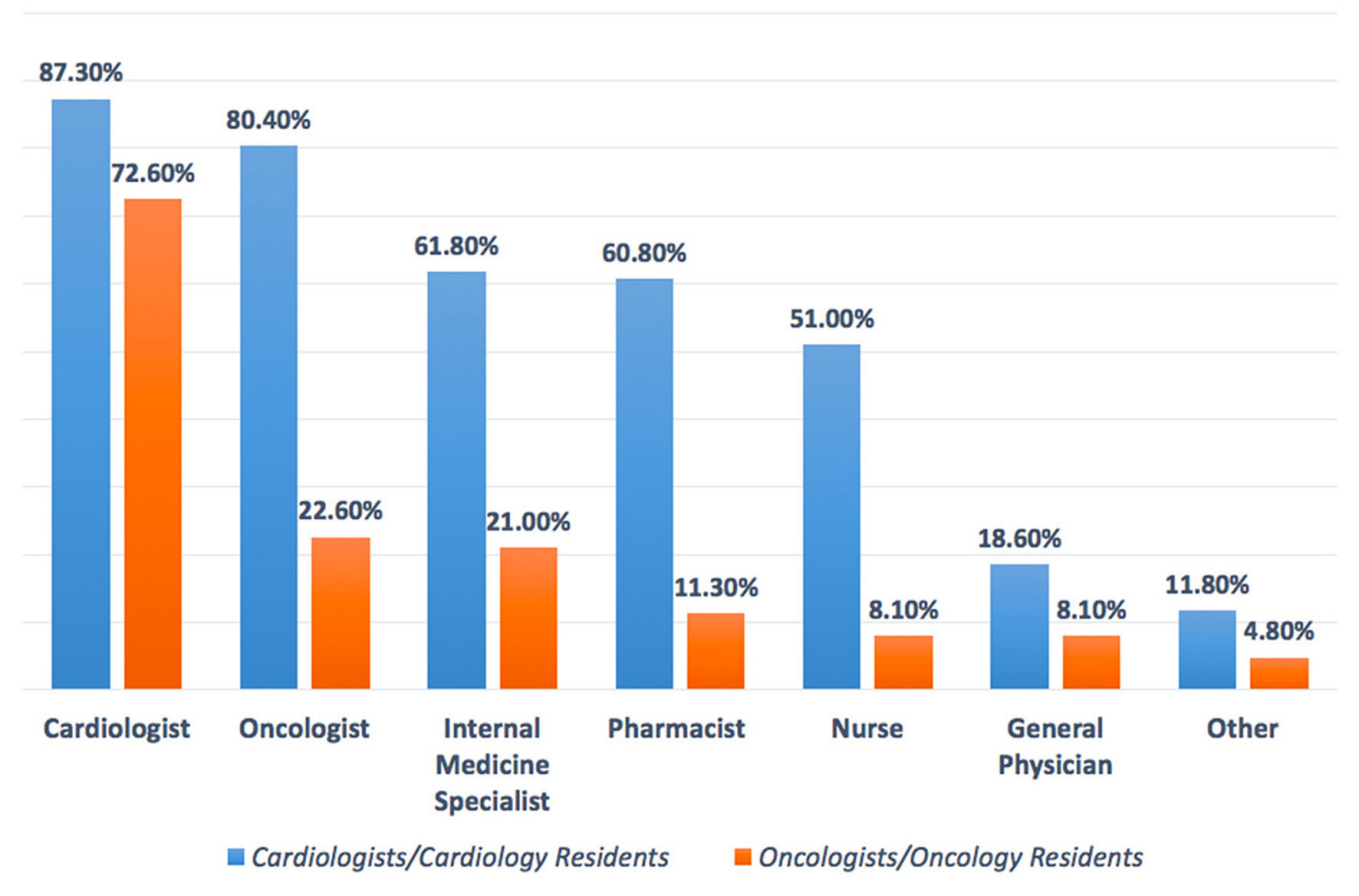
Suggest cardio-oncology team members. Cardiologists, oncologists, internal medicine specialists, and pharmacists are the main specialists in the cardio-oncology team. Oncologists/oncology residents tend to focus mainly on cardiologists as a member of the cardio-oncology team.

The majority of CCRs (86.3%) and OORs (85.5%) believe that the establishment of cardio-oncology services will improve the outcomes of patients. The details are available in (Table 3 and Figure 8).

**Table 3:** Questions about cardio-oncology service.

1. For cardio-oncology teamwork for the management of cancer patient, which specialties do you think it is important to be available? (please choose all options you think it is mandatory)	The main selected specialties are shown in (Figure 8), with cardiologist being the highest recommended expert to be in the team as responding by CCRs (87.3%) and OORs (72.6%). Other specialties suggested, namely: surgeon, hematologist, radiologist, nuclear medicine specialist, nephrologist, psychologist, pulmonologist, nutritionist, and echocardiographer.
2. Do you think it is important to establish a cardio-oncology service for better outcomes of patients with cancer?	Most of CCRs (86.3%) and OORs (85.5%) believe it is important to establish cardio-oncology service, (12.8%) and (14.5%) of CCRs and OORs; respectively, think it is maybe important to establish such service, while only (1%) of CCRs do not think it is important.
3. If there is a plan to establish cardio-oncology service, where is the best place for it?	Most of CCRs (76.5%) and OORs (66.1%) suggest that the best place for cardio-oncology service to be at the academic center rather than a community center, and regarding the best site for this service, (75.8%) of OORs suggested being at oncology site while (52%) of CCRs preferred to be at a cardiology site.
4. Do you have the interest to work in a cardio-oncology service?	Responses with interest to work in this service including 62.8% of CCRs and 64.5% of OORs.
5. Do you think it is important to include cardio-oncology training among the training curriculum of cardiology/oncology fellowship?	For CCRs, 69.6% of them believe it is important to include cardio-oncology training in the cardiology curriculum, a higher percentage was found among OORs (91.9%) to include such training in the oncology curriculum. Disagreement for including this training was the response of 3.9% of CCRs and 8.1% of OORs. The remaining response from CCRs (26.5%) thinks it may be important to include it in the curriculum.
6. Do you think it is essential to hold a monthly cardio-oncology meeting to discuss challenging cases of heart disease in patients with cancer?	Most CCRs (74.5%) and OORs (90.3%) agreed with the holding of a monthly meeting, (25.5%) of CCRs think it may be essential to hold such meeting, while (9.7%) of OORs find it is not essential.
7. How often do you need the availability of echocardiography and ECG minimally per week for the assessment of patients with cancer?^*^	Most of OORs' response (40.3%) was 2 days/week, (32.3%) need them once weekly, (9.7%) daily, (8.1%) believe their availability is not necessary, (4.8%) as required, (3.2%) once monthly, and (1.6%) three times monthly.

* *Indicating this question was directed only for OORs*.

*CCR, cardiologists/cardiology residents; OORs, oncologists/oncology residents*.

## Discussion and Conclusion

This survey showed that HTN was the most common preexisting CVD among patients with cancer according to the response of about two-third of OORs, while none of the CCRs mentioned it as a baseline CVD, at the same time OORs reported prescribing angiotensin-converting enzyme inhibitor/angiotensin receptor blocker (ACEI/ARB) mainly for patients with cancer, this may indicate that they are treating HTN in such patients without referring to cardiologists. Published evidence documented that HTN is the most common baseline comorbid disease among patients with cancer with the prevalence of 38–42% before initiation of cancer therapy, however, after initiation of cancer therapy the incidence of *de novo* or worsening HTN ranging between 17 and 80% (6–9). It is known that CVD and cancer are sharing the same risk factors and preventive strategies, and pre-existing CVD in newly diagnosed patients with cancer may attribute to increasing morbidity and mortality, however, limited studies focus light on the prevalence of pretreatment cardiovascular risk factors (10, 11). A recently published JACC perspective can be considered as a roadmap to improve education and training in the field of cardio-oncology to prevent CVD among patients with cancer rather than direct treatment (12). Regarding the most common CVD induced by chemotherapy and radiation therapy, both the CCRs and OORs reported HF as the first complication, then CCRs reported HTN as the second cardiac complication followed by myocardial infarction, while OORs reported the second complication is myocardial infarction followed by HTN, therefore, both the CCRs and OORs confirm that HTN incidence is among the first top three complications of cancer therapy. HF is a known complication of cancer and radiation therapies particularly in breast cancer, in addition, chest radiation accelerates atherosclerosis and cardiomyopathies (2, 13–15). Systemic HTN can be associated with both chemotherapies and immunologic therapies thus further increasing the burden of cardiovascular toxicities, it was estimated that novel cancer therapy can induce HTN by 24% (7–13). Moreover, resistant HTN and hypertensive crisis are associated with surgery or radiotherapy involving the head or neck (8). Therefore, treatment of HTN is one of the cornerstones for reducing the major cardiovascular events such as HF and end-stage renal failure in addition to overall mortality (7). The most common cardiovascular drug prescribed was ACEI/ARB as reported by both CCRs and OORs followed by anticoagulant and aspirin prescribed by OORs and beta-blockers prescribed by CCRs, this may be interpreted by the OORs use of ACEI/ARB for treating HTN among patients with cancer as a first-line drug as mentioned above. While ACEI/ARN and BB explain that CCRs prescribe them for patients with cancer who are (at risk) of CVD and patients with HF. In UK-based cardio-oncology service, it was also documented that beta-blockers and ACEI/ARB were the most prescribed drug for the referred cardio-oncology patients (6). Beta-blockers are considered cardioprotective drugs among patients with cancer for their beneficial effect in CVD and for the growing evidence regarding their role in breast cancer as it is associated with a significantly lower rate of metastasis particularly with propranolol, significantly reducing HF incidence during anthracyclines therapy with or without trastuzumab, and reducing of cancer-specific mortality rate among patients with prostate cancer whether using cardioselective or noncardioselective beta-blockers (16). Most of the CCRs and OORs reported that the main cancer type associated with CVD related to chemotherapy and radiation therapy are breast cancer and lung cancer, therefore, focusing attention on these two types of cancer particularly baseline risk factors assessment is important to prevent CVD complications. Current evidence also reported breast cancer as the first type of malignancies associated with CVD and the cause of referral (6, 14). More than two-third of OORs reported facing drug-drug interactions, despite this, they are checking for drug-drug interaction only when it is required depending on symptoms as reported by more than two-third of OORs and by about half of CCRs, i.e., checking after the interactions were taking place and this will increase the incidence of drug-drug interactions. Documented harmful drug-drug interactions among patients with cancer who are receiving oral cancer therapy are very common reaching 46% including 15% major and 83% moderate harmful interactions, 14% of interactions including cancer therapy and QT interactions (17). According to the OORs response of survey, CVD is the sixth complication of chemotherapy, however, the majority of the response of OORs regarding the main cause of hospitalization and mortality among cardio-oncology patients is CVD, this may be explained by uncontrolled baseline CV risk factors and underestimating of evaluation drug-drug interactions which may increase CVD or risks which exacerbate CVD leading to hospitalization and mortality. Evidence showed that cardiovascular hospitalization is the main cause of prostate cancer and the third cause of noncancer hospitalization among patients with cancer in general and noncancer mortality is highest among patients with colorectal, bladder, kidney, endometrium, breast, prostate, and testis cancers with heart disease being the most common cause (18, 19). Other findings in this study reported more than two-third of CCRs and OORs are facing difficulties while taking management action plans among cardio-oncology patients which necessitates teamwork and availability of guidelines and protocols for the management of cardio-oncology patients. Most CCRs and OORs agreed that the cardio-oncology team should include cardiologists, however, most CCRs believe that the team should include also oncologists, internal medicine specialists, and pharmacists, in addition to other specialties, however, the minority of OORs tend to include other specialties among the cardio-oncology team. It is known that cardiologists have an important role in the prevention of cardiovascular complications among patients with cancer through a comprehensive evaluation of cardiovascular risk factors and physical examination before initiation of cancer therapy (20), however, other specialties are also essential to be included among the cardio-oncology team for better outcomes, for example, the availability of pharmacists to prevent or minimize harmful drug-drug interactions which commonly occur (17). The proposed typical cardio-oncology team includes cardiologists, oncologists, hematologists, general practitioners, pharmacists, nurses, cardiac surgeons, radiologists, clinical laboratory specialists, palliative care team, psychologists, social workers, and data managers, depending on hospital size and organization (20). The current survey discovered several encouraging and strength points for the initiation of cardio-oncology services in Iraq including more than one-half of both CCRs and OORs have the interest to work in cardio-oncology service, most of the CCRs and OORs believe in the importance of holding a monthly cardio-oncology meeting to discuss together challenging cardio-oncology cases, and two-third of CCRs want to include cardio-oncology training in cardiology curriculum and almost all OORs wish to include such training in the oncology curriculum, all these points are promising for the near future improvement in the standard of cardiac services for patients with cancer to achieve the mission of our cardio-oncology program by protecting heart health among cardio-oncology patients.

This study depended on an online survey reflecting the expert opinions, therefore, the real prevalence of baseline cardiovascular risk factors and other results among patients with cancer need to be documented by clinical researchers and registries.

In conclusion, according to the survey-based data depending on the perception of cardiologists and oncologists, CVD is the leading cause of hospitalization and mortality among cardio-oncology patients, despite that CVD may be considered as the sixth rank complications among cancer therapy. There are essential needs to focus on the baseline CV risk stratification among patients with cancer to prevent CVD or CVD worsening by emphasizing on teamwork. There is an increasing interest among cardiologists, oncologists, and their residents in cardio-oncology teamwork and cardio-oncology training. It is time to take the action plan to change the current real practice to bridge the gap in the cardio-oncology service and to support clinical researches and registries in this field.

## Data Availability Statement

The raw data supporting the conclusions of this article will be made available by the authors, without undue reservation.

## Author Contributions

HF and IY contributed equally in study design, writing, review, and approve for publication. IY performed the statistical analysis.

## Conflict of Interest

The authors declare that the research was conducted in the absence of any commercial or financial relationships that could be construed as a potential conflict of interest.

## Publisher's Note

All claims expressed in this article are solely those of the authors and do not necessarily represent those of their affiliated organizations, or those of the publisher, the editors and the reviewers. Any product that may be evaluated in this article, or claim that may be made by its manufacturer, is not guaranteed or endorsed by the publisher.
